# Hippocampal phase precession may be generated by chimera dynamics

**DOI:** 10.3389/fncir.2025.1634298

**Published:** 2025-10-06

**Authors:** Maria Masoliver, Jörn Davidsen, Wilten Nicola

**Affiliations:** ^1^Department of Physics and Astronomy, University of Calgary, Calgary, AB, Canada; ^2^Department of Cell Biology and Anatomy, University of Calgary, Calgary, AB, Canada; ^3^Hotchkiss Brain Institute, University of Calgary, Calgary, AB, Canada

**Keywords:** hippocampus, phase precession, chimera states, non-linear dynamics, oscillations, partial synchronization

## Abstract

The 8 Hz theta rhythm observed in hippocampal local field potentials of animals can be regarded as a “clock” that regulates the timing of spikes. While different interneuron sub-types synchronously phase lock to different phases for every theta cycle, the phase of pyramidal neurons' spikes asynchronously vary in each theta cycle, depending on the animal's position. On the other hand, pyramidal neurons tend to fire slightly faster than the theta oscillation in what is termed hippocampal phase precession. Chimera states are specific solutions to dynamical systems where synchrony and asynchrony coexist, similar to coexistence of phase precessing and phase locked cells during the hippocampal theta oscillation. Here, we test the hypothesis that the hippocampal phase precession emerges from chimera dynamics with computational modeling. We utilized multiple network topologies and sizes of Kuramoto oscillator networks that are known to collectively display chimera dynamics. We found that by changing the oscillators' intrinsic frequency, the frequency ratio between the synchronized and unsynchronized oscillators can match the frequency ratio between the hippocampal theta oscillation (≈ 8 Hz) and phase precessing pyramidal neurons (≈ 9 Hz). The faster firing population of oscillators also displays theta-sequence-like behavior and phase precession. Finally, we trained networks of spiking integrate-and-fire neurons to output a chimera state by using the Kuramoto-chimera system as a dynamical supervisor. We found that the firing times of subsets of individual neurons display phase precession.

## Introduction

The hippocampus executes a complex dynamical repertoire across spatial and temporal scales to aid in behaviors that are critical for survival such as memory formation (Hasselmo et al., 2002; Manns et al., 2007; Siegle and Wilson, 2014; Hasselmo, 2005; Pastalkova et al., 2008; Wang et al., 2015; Diba and Buzsáki, 2007; Buzsáki, 1989, 2002; Hasselmo and Stern, 2014) and navigation (Hasselmo and Stern, 2014; O'keefe and Burgess, 2005; King et al., 1998; Ego-Stengel and Wilson, 2007; Dragoi et al., 1999; O'Keefe and Dostrovsky, 1971; Lee and Wilson, 2002). For example, the observed 8 Hz (theta) oscillation in the local field potential organizes spikes across space (Davidson et al., 2009; Mamad et al., 2015; Cei et al., 2014; Ego-Stengel and Wilson, 2007), time (Salz et al., 2016), behavior (Bender et al., 2015; Boyce et al., 2016; Cei et al., 2014; Hasselmo et al., 2002; Manns et al., 2007; Kunec et al., 2005), neuronal populations (Klausberger and Somogyi, 2008; Klausberger et al., 2004; Amilhon et al., 2015; Klausberger et al., 2003; Lapray et al., 2012; Somogyi and Klausberger, 2005), and hippocampal anatomy (Lubenov and Siapas, 2009).

Behaviourally, the theta oscillation is observed in mice and rats when they are actively engaged in memory or navigational tasks, or during Rapid Eye Movement (REM) sleep (Heusser et al., 2016; Skaggs et al., 1996; O'Keefe and Recce, 1993). The theta oscillation is critical for memory formation during these task as optogenetic or pharmacological perturbation can disrupt subsequent recall (Pastalkova et al., 2008; Wang et al., 2015). At the spatial level, the theta oscillation acts as a traveling wave across the septo-temporal axis of the hippocampus. Depending on the specific interneuron sub-type, interneurons primarily lock their spike times to different phases of the hippocampal theta oscillation (Klausberger and Somogyi, 2008; Klausberger et al., 2004; Amilhon et al., 2015; Klausberger et al., 2003; Lapray et al., 2012; Somogyi and Klausberger, 2005). Pyramidal neurons, however, fire slightly faster than the hippocampal theta oscillation, by approximately 1 Hz (O'Keefe and Recce, 1993; Skaggs et al., 1996; Pastalkova et al., 2008). This frequency difference results in an effect called hippocampal phase precession, where the phase of the pyramidal neuron decreases on successive cycles.

Due to its importance in organizing hippocampal dynamics and organism behaviors across scales, the origins and mechanisms of the hippocampal theta oscillation and hippocampal phase precession have been intensely studied and subsequently debated (Buzsáki, 2002; Skaggs et al., 1996; O'Keefe and Recce, 1993; Hasselmo and Stern, 2014; Hasselmo et al., 2002; Manns et al., 2007; Kunec et al., 2005; Hasselmo et al., 1996; Siegle and Wilson, 2014; Hasselmo, 2005; Nicola and Clopath, 2019; Ferguson et al., 2017, 2015; Lubenov and Siapas, 2009; Boyce et al., 2016; Amilhon et al., 2015). The oscillation itself may be extra-hippocampal, as perturbations to the medial septum in the diagonal band of Broca lead to direct changes in the hippocampal theta oscillation. Lesioning (Lee et al., 1994), or pharmacological inhibition (Wang et al., 2015) of the medial septum reduces the power of or eliminates the hippocampal theta oscillation while other manipulations to the medial septum can alter the theta oscillation frequency (Petersen and Buzsáki, 2020; Bender et al., 2015; Zutshi et al., 2018). However, the whole isolated hippocampus or suitably large hippocampal slices can autonomously produce the hippocampal theta oscillation (Goutagny et al., 2009). Computational modeling has shown this is possibly due to a subset of pacemaker neurons coupled with recurrent excitation, or, alternatively, as an emergent dynamical state through inhibitory neuronal interactions, or potentially emergent through local excitatory/inhibitory interactions (Ferguson et al., 2017, 2015; Chatzikalymniou and Skinner, 2018; Nicola and Clopath, 2019; Chadwick et al., 2016; Tsodyks et al., 1996; Bose et al., 2000). Some models additionally postulate that hippocampal phase precession is inherited from other areas (Jaramillo et al., 2014), or created by short-term plasticity effects (Thurley et al., 2008), while other models explicitly analyze how theta-gamma coupling emerges in neural circuits (Vardalakis et al., 2024; Scheffer-Teixeira and Tort, 2016)

In this work, rather than analyzing the network topology or biophysical mechanism of the hippocampal theta oscillation, we instead investigate the class of dynamics that can produce phase precession in coupled oscillator systems. While a straightforward oscillation as a limit cycle is one possibility, the simultaneous existence of synchronized phase-locked subpopulations of interneurons and asynchronous phase advancing pyramidal cells points to more complex dynamics. Thus, we consider chimera states, where synchronized and unsynchronized populations of oscillators co-exist (Kuramoto and Battogtokh, 2002; Abrams and Strogatz, 2004; Davidsen, 2018; Parastesh et al., 2020), as the dynamical state responsible for the theta oscillation's diverse repertoire. Chimera's emerge as specific solutions in non-linear dynamical systems where subsets of nodes synchronize onto a common solution, while other subsets display an asynchronous state despite all units being either explicitly identical or drawn from identical heterogeneous distributions.

**Figure 1:**
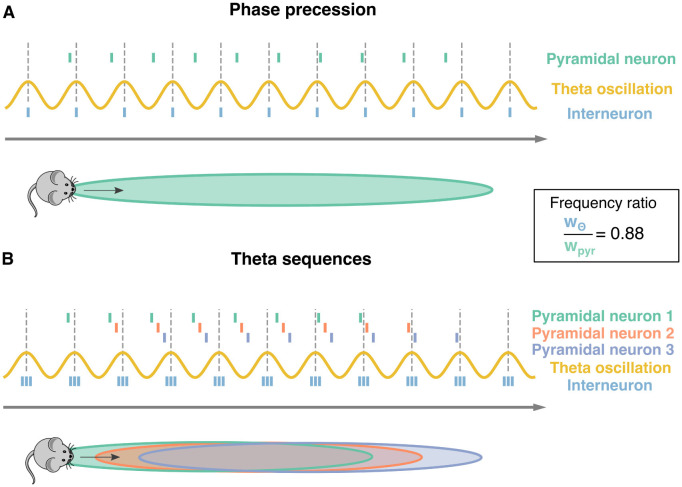
Phase precession and theta sequences. Schematic representation of phase precession and theta sequences. Dotted lines: peaks of the theta oscillation (yellow sinusoidal curve). The distance between two subsequent lines defines one theta cycle. **(A)** As a mouse moves along a track (gray arrow); a pyramidal neuron starts firing as the animal enters the pyramidal neurons' place field (green surface). While the actions potentials from the pyramidal neuron (green ticks) happen earlier at each theta cycle, the ones from an interneuron (blue ticks) are usually synchronized to the theta oscillation. This phase advancement from the theta cycle is known as phase precession. The ratio between theta oscillation's frequency and pyramidal neuron's frequency is approximately 0.88 = 8 Hz/9 Hz as pyramidal cells tend to fire at approximately 1 Hz faster than the 8 Hz theta oscillation. **(B)** Three pyramidal neurons undergoing phase precession and their corresponding place fields are considered (green, red, and purple). Having multiple neurons leads to sequences of spikes within a theta oscillation, known as theta sequences. Three interneurons (blue ticks) are depicted as well.

To test the hypothesis that hippocampal phase precession is a chimera state, we utilized existing computational models of chimera dynamics. The first set of models consisted of Kuramoto oscillators coupled with multiple network topologies that all yielded chimera dynamics (Kuramoto and Battogtokh, 2002; Abrams and Strogatz, 2004, 2006; Laing, 2009). We found that by changing the oscillators' intrinsic oscillation frequency, the frequency ratio between the synchronized and unsynchronized oscillators can match the frequency ratio between interneurons and pyramidal neurons in the hippocampus. The unsynchronized oscillators oscillate approximately 1 Hz faster, as seen in the pyramidal neurons undergoing phase precession (see Figure 1). These unsynchronized populations of oscillators also display sequential activity on a longer-time scale, similar to pyramidal neurons in the hippocampus during active navigation (see Figure 1). Finally, we considered more biologically plausible models to investigate if the chimera state is responsible for hippocampal phase precession. We trained networks of spiking Izhikevich neurons (Izhikevich, 2003) to output a chimera state by using a Kuramoto-chimera system as a dynamical supervisor with FORCE training (Sussillo and Abbott, 2009; Nicola and Clopath, 2017). We found that the firing times of subsets of individual neurons display phase precession and long time scale spike sequences. These results imply that the hippocampal phase precession may be a chimera state, further suggesting the importance of chimera states in neuroscience.

## Methods

###  Chimera on a ring

The chimera state on a ring was obtained from the integration of *N* Kuramoto oscillators with nonlocal coupling. The equations are given by Abrams and Strogatz (2006):


(1) $$\begin{array}{l} \frac{d\phi_{i}}{dt}=\rho -\frac{1}{N}\overset{N}{\underset{j=1}{\sum }}\left[1+A\cos\left(\frac{2\pi |i-j|}{N}\right)\right]\cos\left(\phi_{i}-\phi_{j}-\beta \right) \end{array}$$

with *i* = 1, ..., *N*, where ϕ_*i*_ is the oscillator's phase and ρ is the oscillators intrinsic frequency. To obtain a stable chimera we integrated Equation 1 using chimera-like initial conditions and we set *A* = 0.95, β = 0.2 and *N* = 500 as in Laing (2009). We chose *A* and β from the (*A*, β) parameter plane in which the chimera state exists (see ref. Abrams and Strogatz (2006) for details) and set *N* large enough (*N*>50) such that for this type of network the chimera did not collapse (note that the number of oscillators can be reduced when using a different topology as the one used in Equation 1, see ref. Panaggio et al. (2016) for examples). We obtained chimera-like initial conditions by randomly selecting the same phase for half of the network. The phases for the other half were selected from a uniform distribution between [0, 2π]. See ref. Masoliver et al. (2022) for more details. The equations were integrated using the Euler method with an integration step of *dt* = 10^−3^. Note that all equations are dimensionless, however time can be rescaled so that a single unit of time, which corresponds to 8 cycles of the synchronized population, can be rescaled to 1 second which yields an 8 Hz (theta) oscillation.

###  Two-population chimera

The two-population chimera consists of two populations of *n* Kuramoto oscillators each. The phases of the oscillators for group 1 and group 2 are given by $\gamma ={\left\{\gamma_{i}\right\}}_{i=1}^{n}$ and $\phi ={\left\{\phi_{i}\right\}}_{i=1}^{n}$, which are governed by the following equations:


(2) $$\begin{array}{l} \tau \frac{d\gamma_{i}}{dt}=\rho -\mu \overset{n}{\underset{j=1}{\sum }}\cos\left(\gamma_{i}-\gamma_{j}-\beta \right)-\nu \overset{n}{\underset{j=1}{\sum }}\cos\left(\gamma_{i}-\phi_{j}-\beta \right) \end{array}$$

(3) $$\begin{array}{l} \tau \frac{d\phi_{i}}{dt}=\rho -\mu \overset{n}{\underset{j=1}{\sum }}\cos\left(\phi_{i}-\phi_{j}-\beta \right)-\nu \overset{n}{\underset{j=1}{\sum }}\cos\left(\phi_{i}-\gamma_{j}-\beta \right). \end{array}$$

**Figure 2:**
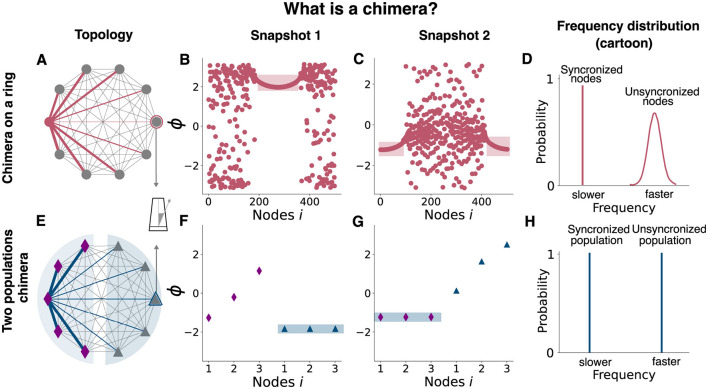
Chimera states in two networks of Kuramoto Oscillators. Schematic representation of two different chimera states: the chimera on a ring (top) and the two-population chimera. For both topologies, each node is a Kuramoto Oscillator. **(A)** Diagram showing the coupling scheme needed to observe a chimera on a ring. A non-local coupling rule is used, see Equation 1 for details. For clarity, only the coupling for a single node or oscillator (pink node) has been depicted (pink edges). Edge thickness represents the weights of a connection. **(B)** Snapshot of oscillators' phases at a given time. Light pink rectangle denotes the synchronized nodes. **(C)** Snapshot of oscillators' phases at a different time. Light pink rectangle denotes the synchronized nodes. **(D)** Schematic representation (cartoon) of the ring oscillators' frequency distribution. The synchronized nodes oscillate at the same frequency (within them) but at a slower pace than the unsynchronized ones. **(E)** Diagram showing the coupling scheme needed to observe a chimera on two populations: two populations (diamonds and triangles) are weakly coupled between each other and strongly coupled within, see Equations 2, 3 for details. For clarity, just the coupling for one node (blue) has been depicted (blue). Edge thickness represents the weights of its connection. **(F)** Snapshot of oscillators' phases at a given time. Light blue rectangle marks the synchronized population. **(G)** Snapshot of oscillators' phases using different initial conditions or after the system is externally perturbed (Figure 4). Light blue rectangle marks the synchronized population. **(H)** Schematic representation of the ring oscillators' frequency distribution. The synchronized population oscillates at a slower pace than the unsynchronized one. The nodes for each population oscillate at the same frequency.

**Figure 3:**
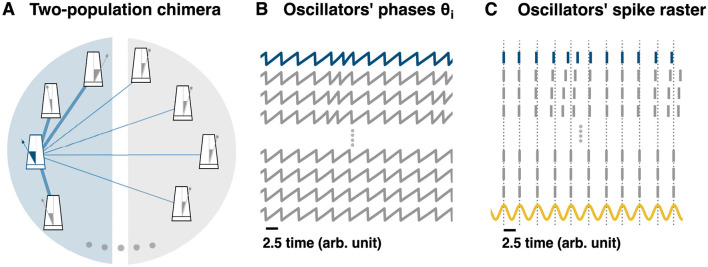
From a two-population chimera state to hippocampal phase precession. **(A)** Schematic representation of a two-population topology induced chimera. Non-local coupling is used, see main text for equations. For clarity, only the coupling for the blue oscillator (blue or dark gray metronome for b/w printing) has been depicted (blue or dark gray edges for b/w printing). Edge thickness represents the connection weight strength. **(B)** Time-series of different oscillators: some unsynchronized and some synchronized. **(C)** Oscillators' spike raster plot: **(B)** transformed into a raster plot. See Methods (main text) for details. The sinusoidal curve (yellow) represents the theta oscillation. It is computed as cos(ϕ_*j*_), where ϕ_*j*_ corresponds to the phase from any of the synchronized oscillators. Dotted lines correspond to the peaks of the sinusoidal signal and to the spikes of the synchronized nodes.

The coupling within groups is given by $\mu =\frac{1+A}{2n}$ and between groups by $\nu =\frac{1-A}{2n}$, with ν < μ, 0 ≤ *A* ≤ 1 and *n* = 3. To obtain a stable chimera, we simulated Equations 2 and 3 with appropriate initial conditions as in ref. Masoliver et al. (2022) and we fixed β = 0.025 and *A* = 0.1. The temporal component $\tau =\frac{1}{0.012}$ is used to slow down the chimera dynamics, which is needed in order to successfully train the spiking recurrent network. It is for that value that we get 8 and 9 oscillations, for the synchronized and the unsynchronized populations, respectively, in 1 second. Figures 2E, 3A illustrate the network. The equations were integrated using the Euler method with an integration step of *dt* = 10^−3^. Note that all equations are dimensionless, but can be rescaled in time as described in the chimera-on-a-ring case.

###  Noise

To investigate the robustness of the chimera state(s) to noise (in Figure 11), an uncorrelated white noise term was added to each oscillator for the chimera on a ring:


(4) $$\begin{array}{l} \frac{d\phi_{i}}{dt}=\rho -\frac{1}{N}\overset{N}{\underset{j=1}{\sum }}\left[1+A\cos\left(\frac{2\pi |i-j|}{N}\right)\right]\\ \;\times \cos\left(\phi_{i}-\phi_{j}-\beta \right)+\zeta_{i}\left(t\right) \end{array}$$

where ζ_*i*_(*t*) has mean 0, and standard deviation σ.

###  From phases to spikes

Since the oscillators are periodic and oscillate between 0 and 2π, we can transform the oscillators' time-series (Figures 2B, 3A) into a raster plot. Every time the oscillator's phase ϕ_*i*_ = 0 a spike is drawn. The resulting raster plot from the aforementioned time-series is shown in for the chimera on a ring and in Figure 3C for the two populations chimera.

###  Mean phase velocity

The mean phase velocity (Omelchenko et al., 2013) for a given oscillator with phase θ_*i*_ is defined as :


(5) $$\begin{array}{l} \Omega_{i}=\frac{2\pi M_{i}}{\Delta t}, \end{array}$$

where *M*_*i*_ is the number of complete rotations around the origin performed by the *i*th oscillator during the time interval Δ*t* = 1000. It acts as a measure of the oscillating frequency for each oscillator. Given a ring of *N* oscillators, it is denoted as Ω = Ω_*i*_, with *i* = 1, 2, …*N*. Having different mean phase velocities for the synchronized and unsynchronized domain is typical for chimera states. In particular, for ring-like topologies it is common to have an arc-like profile of mean phase velocities for the unsynchronized domain, denoted as $\Omega^{u}=\Omega_{j}^{u},\ldots \Omega_{m}^{u}$ (where *u* stands for unsynchronized and *m* is the total number of unsynchronized oscillators) and equal mean phase velocities for the synchronized domain (Gu et al., 2013; Kemeth et al., 2016; Sawicki et al., 2017) given by $\Omega^{s}=\Omega_{j}^{s},\ldots \Omega_{n}^{s}$ (where *s* stands for synchronized and *n* is the total number of synchronized oscillators). Since the oscillators are synchronized they have the same mean phase velocity Ω^*s*^, therefore we can simplify Ω^*s*^ to a unique value given by Ω_*s*_. We will use Ω_*s*_ to identify which oscillators synchronize and which do not, given that the synchronized domain oscillates at a slower pace than for the unsynchronized one Ω^*s*^ < Ω^*u*^ ∀*j*∈*m*. In order to identify Ω^*s*^, one can simply compute the minimum of Ω.

For the two-population chimera, both domains (synchronized and unsynchronized) have equal mean phase velocities (different between domains but equal within). Also for that topology, the synchronized population oscillates at a slower pace than for the unsynchronized one: Ω^*s*^ < Ω^*u*^.

###  Mean phase velocity ratio

#### Chimera on a ring

For each intrinsic frequency ρ we compute the mean phase velocity ratio, which measures the relation between the mean phase velocity of the synchronized domain vs. the unsynchronized one. We define the mean velocity ratio as follows:


$$\begin{array}{l} {\langle \Omega \rangle }_{ratio}=\frac{1}{m}\overset{j=m}{\underset{j=0}{\sum }}\frac{\Omega^{s}}{\Omega_{j}^{u}} \end{array}$$

and the standard deviation as:


$$\begin{array}{l} \sigma =\sqrt{\frac{1}{m}\overset{j=m}{\underset{j=0}{\sum }}{\left(\frac{\Omega^{s}}{\Omega_{j}^{u}}-{\langle \Omega \rangle }_{ratio}\right)}^{2}} \end{array}$$

We note that the mean-phase velocity ratio is a dimensionless quantity.

#### Two-population chimera

For the two-population chimera, the mean phase velocity is simplified, since we do not have a unique value only for Ω^*s*^ but also for Ω^*u*^. The mean phase velocity is:


(6) $$\begin{array}{l} \Omega_{ratio}=\frac{\Omega^{s}}{\Omega^{u}} \end{array}$$

since we do not have a set of values for Ω_*u*_, there is no variation when computing Ω_*ratio*_ as depicted.

###  Spiking neural network equations and the FORCE method

The spiking neural network consists of coupled Izhikevich neurons (Izhikevich, 2003), with their dynamics given by the following equations:


(7) $$\begin{array}{l} C\frac{dv_{i}}{dt}=k\left(v_{i}-v_{r}\right)\left(v_{i}-v_{t}\right)-u_{i}+I_{i} \end{array}$$

(8) $$\begin{array}{l} \frac{du_{i}}{dt}=a\left(b\left(v_{i}-v_{r}\right)-u_{i}\right). \end{array}$$

The quantity *v*_*i*_ is the voltage variable. Neuron *i* fires a spike when *v*_*i*_ reaches a voltage peak *v*_*peak*_ and it is instantly reset to a potential *v*_*reset*_. The adaptation current is given by *u*_*i*_, which increases an amount *d*_*u*_ every time a spike is fired and which in turn slows down the production of spikes. The current *I*_*i*_ is given by *I*_*i*_ = *I*_*bias*_+*s*_*i*_, where *I*_*bias*_ is a fixed value and *s*_*i*_ are the synaptic currents for neuron *i*, given by


(9) $$\begin{array}{l} s_{i}=\overset{N}{\underset{j=1}{\sum }}\omega_{ij}^{0}r_{j}, \end{array}$$

where *N* is the total number of neurons. The matrix $\omega_{ij}^{0}$ controls the magnitude of the postsynaptic currents arriving at neuron *i* from neuron *j*. The parameter *C* represents the membrane capacitance, the parameters *v*_*r*_ and *v*_*t*_ denote the resting and the threshold membrane potential, respectively. The parameter *a* is an equivalent of the time constant for the adaptation current *u*_*i*_. The parameter *b* controls the resonance properties of the model and *k* controls the half-width of the action potentials. The numeric parameters of the model are listed in Table 1, we used the same parameters as in Nicola and Clopath (2017). The spikes are filtered with a double exponential synapse, given by:


(10) $$\begin{array}{l} \frac{dr_{j}}{dt}=-\frac{r_{j}}{\tau_{d}}+h_{j} \end{array}$$

(11) $$\begin{array}{l} \frac{dh_{j}}{dt}=-\frac{h_{j}}{\tau_{r}}+\frac{1}{\tau_{r}\tau_{d}}\underset{t_{jk<t}}{\sum }\delta \left(t-t_{jk}\right), \end{array}$$

where τ_*r*_ = 2 ms is the synaptic rise time, τ_*d*_ = 20 ms is the synaptic decay time and *t*_*jk*_ is the time at which the neuron *j*th fired spike *k*th. For other synapse types, see Nicola and Clopath (2017).

**Table 1:** Neural parameters used to train the spiking neural network, described in Equations 7, 8, 14.

**Parameter**	**Value**
*N*	10,000
*C*	250 μF
*v*_*peak*_	30 mV
*v*_*reset*_	–65 mV
*d*_*u*_	200 mV
*I*_*bias*_	1,000 pA
*v*_*r*_	–60 mV
*v*_*t*_	–20 mV
*a*	0.01 ms^−1^
*b*	–2 ns
*k*	2.5 ns/mV
*G*	15,000
*Q*	1,400

The output of a spiking neural network is defined as:


(12) $$\begin{array}{c} \hat{x}=\overset{N}{\underset{j=1}{\sum }}d_{j}r_{j}, \end{array}$$

where ***d***_*j*_ is an *m*-dimensional vector known as the linear decoder for the firing rate. Here, we want to train the network such that:


(13) $$\begin{array}{c} \hat{x}\left(t\right)\approx x\left(t\right) \end{array}$$

where *x* = (*x*_1_, *x*_2_, …, *x*_*m*_) are the desired dynamics or the supervisor that the network should mimic. Since the oscillators' phases γ and ϕ are discontinuous and wrapped around the interval [0, 2π), the following supervisor for the chimera was used: *x* = (cosϕ, sinϕ, cosγ, sinγ). With 2*n* (*n* = 3) oscillators, this results in a *m* = 4*n* = 12 dimensional supervisor, see Masoliver et al. (2022) for details.

In order to achieve Equation 13 we use the FORCE method (Sussillo and Abbott, 2009), which adds a second set of weights $Q\eta_{i}\cdot d_{j}^{T}$ when defining the synaptic currents. Equation 9 can be rewritten as:


(14) $$\begin{array}{c} s_{i}=\overset{N}{\underset{j=1}{\sum }}\left(G\omega_{ij}^{0}+Q\eta_{i}\cdot d_{j}^{T}\right)r_{j} \end{array}$$

(15) $$\begin{array}{cc} = & \overset{N}{\underset{j=1}{\sum }}G\omega_{ij}^{0}r_{j}+Q\eta_{i}\hat{x} \end{array}$$

The FORCE method has three phases, the pre-learning, the learning and the post-learning. In the pre-learning phase, the initial synaptic connection matrix $w_{ij}^{0}$ initializes the neurons' dynamics into a well-known high-dimensional chaotic regime (Ostojic, 2014; Harish and Hansel, 2015). The matrix is static and sparse with each element drawn from a normal distribution with mean 0 and variance $\frac{1}{Np^{2}}$, where *p* is the sparsity degree (set to 90% sparse or *p* = 0.1). The variable *G* controls the network's chaotic behavior and its value depends on the neuronal model, see Nicola and Clopath (2017) for a detailed explanation. Here, we set *G* = 1.5 × 10^3^.

The learning phase involves a second set of weights, given by $Q\eta_{i}\cdot d_{j}^{T}$. Where the parameter *Q* scales the encoding vector η_*i*_, which has been drawn randomly and uniformly from [−1, 1]^*m*^ (where *m* is the dimensionality of the supervisor). By increasing *Q*, the feedback applied to the network is strengthened. A value of *Q* = 1.4 × 10^3^ was used for all simulations.

In the learning phase, the FORCE method enforces the aforementioned constrain $\hat{x}\approx x$ by changing ***d**_i_* online (i.e., as the network is being simulated) with the Recursive Least Squares (RLS) (Sussillo and Abbott, 2009). RLS has an online solution for the optimal ***d***, the one that minimizes the squared error **e** between the network output $\hat{\text{x}}$ and the complex signal or supervisor **x**. RLS updates to ***d*** at each time step *n* are:


(16) $$\begin{array}{c} d_{n+1}=d_{n}-P_{n+1}^{-1}r_{n}{\text{e}}_{n} \end{array}$$

(17) $$\begin{array}{c} P_{n+1}^{-1}=P_{n}^{-1}-\frac{P_{n}^{-1}r_{n}{r_{n}}^{⊤}P_{n}^{-1}}{1+{r_{n}}^{⊤}P_{n}^{-1}r_{n}} \end{array}$$

where ***d***_0_ = 0 and *P*_0_ = *I*_*n*_/λ. The parameter λ controls the rate of the error (Sussillo and Abbott, 2009) and we set it to λ = 1. The parameter **I**_*n*_ is a *N*×*N* identity matrix.

The third step of the FORCE method is the post-learning phase. RLS is turned-off and the weight matrix $Q\eta_{i}\cdot d_{j}^{T}$ is no longer dynamic but static. The FORCE method is successful if the network is able to reproduce the supervisor for a fixed ***d***.

Finally, Dale's law can also be enforced in trained spiking neuronal networks. In Dale's law, a neuron can only be either inhibitory or excitatory, not both. Dale's Law was enforced by constraining ω to the inhibitory/excitatory nature of each individual neuron. If neuron *i* is inhibitory (excitatory), all of its outgoing connections will be negative (positive): $\omega_{1i}^{1},\cdots \;,\omega_{Ni}^{1}<0$ ($\omega_{1i}^{1},\cdots \;,\omega_{Ni}^{1}>0$). We first define $\omega_{ij}^{0}$ such that $\omega_{ij}^{0}\geq 0$ ∀*j* ∈[0, *N*] (the first half of the population of neurons only projects positive weights, i.e., excitatory neurons) and $\omega_{ij}^{0}\leq 0$ ∀*j* ∈[0, *N*] (the second half of the population of neurons only projects negative weights, i.e., inhibitory neurons). Second, the trained matrix $Q\eta_{i}\cdot d_{j}^{T}$ is limited to project either positive or negative weights. We obtain that by defining η as η = η_−_+η_+_, where η_−_ and η_+_ are unequivocally defined as negative and positive matrices, respectively. And finally, ***d***^*T*^ is defined such that *d*_*ij*_≥0 $\forall i\in \left[0,\frac{N}{2}\right]$ and *d*_*ij*_ ≤ 0 $\forall i\in \left[\frac{N}{2},N\right]$. For the exact implementation refer to Additional Information where the link to the code is available and for more details see Nicola and Clopath (2019, 2017).

## Results

###  Analyzing existing chimera-inducing network topologies

To investigate if chimera dynamics are a potential mechanism for the neuronal dynamics associated with the hippocampal theta oscillation, chimera dynamics were first simulated in pre-existing models to test the hypothesis that parameter ranges that exhibit hippocampal-like dynamics (i.e., phase precession and sequential content) could readily be determined.

Two standard model versions, each generating a different chimera state—a chimera on a ring and a two-population chimera, respectively—were considered. The chimera on a ring arises for *N* = 500 non-locally coupled identical Kuramoto oscillators (see Figure 2A and Methods, Equation 1), whereas the two-population chimera arises for two weakly coupled populations, each one formed by 3 globally coupled Kuramoto oscillators (see Figures 2E, 3E Methods, Equations 2, 3). Depending on the parameter values and on the initial conditions, both network topologies can display different dynamics: Either a fully synchronized state where all oscillators are in phase or chimera states where one sub-population of neurons is synchronized while the other sub-population oscillates asynchronously (see Supplementary Video S1).

**Figure 4:**
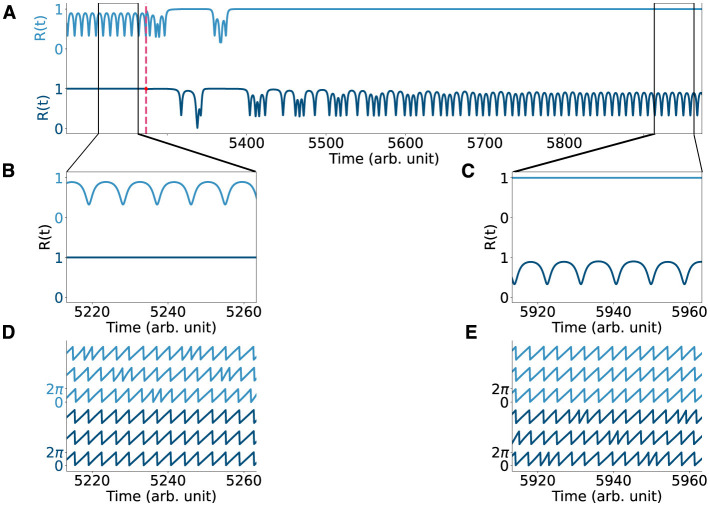
Perturbing the two-population chimera. **(A)** Order parameter R(t) for the two populations chimera **ϕ** (light blue, top) and γ (dark blue, bottom) before and after the system is being perturbed (dashed red line). The order parameter is computed at each time step as $R=|\frac{1}{n}\overset{n}{\underset{j=1}{\sum }}\exp\left(i\theta_{j}\right)|$ and it quantifies the synchronization of any oscillatory system with phases ${\left\{\theta_{i}\right\}}_{i=1}^{n}$. For synchronized systems |*R*| = 1 and for systems that are not fully synchronized, 0 ≤ |*R*| < 1. **(B, C)** Zoom-in of the order parameter before and after the perturbation. **(D, E)** Time-series for the two populations chimera before and after the perturbation.

First, the model parameters of the dynamical equations (Equation 1 and Equations 2, 3, respectively) were set to well known or classical parameter regimes where chimera dynamics readily emerge (Laing, 2009; Panaggio et al., 2016). The parameters *A* and β affect the coupling strength and the phase difference, respectively, and take different values for the two different systems, see Table 2 for details. For the chimera on a ring, the synchronous subpopulation of oscillators is non-static, and drifts slowly around the ring. Oscillators drift in and out of the synchronous sub-population, while the others oscillate asynchronously (see Supplementary Video S2, Figures 2B, C) with a narrow distribution of frequencies (Figure 2D). In contrast, for the two-population chimera, the chimera state is static: one population fully synchronizes (triangles in Figure 2F) while the other one does not (diamonds in Figure 2F). Unless the system is perturbed, the synchronized and unsynchronized populations remain fixed, each with a fixed oscillation frequency (Figure 2H). The identity of the synchronized or unsynchronized population depends on the initial conditions (Figures 2F, G). The synchrony profile between the two populations can be exchanged by externally perturbing the system, where the synchronized and unsynchronized populations swap. For example, in Figure 4, the triangle population is synchronized before a perturbation, and after a perturbation, the oscillators move to an asynchronous regime (vice versa for the diamond population).

**Table 2:** Parameters for the model chimera-on-a-ring, described in Equation 1 and for the two populations chimera, described in Equations 2, 3. For the chimera on a ring, the parameters *A* and β denote the amplitude of the coupling strength and phase offset, respectively, while *N* denotes the number of oscillators.

**Chimera on a ring**	
**Parameter**	**Value**
A	0.95
β	0.2
*N*	500
**Two populations Chimera**	
A	0.1
β	0.025
*n*	3 (varies)
τ	1/0.012
μ	0.18
ν	0.15

For the two population chimera, τ denotes the relative time scale while μ and ν denote the coupling strengths for self-coupling and cross-coupling, respectively. ρ denotes the intrinsic chimera frequency.

For the chimera on a ring, the synchronized and unsynchronized populations drift (Supplementary Videos S1, S2 and Figures 2B, C). An external perturbation, in this case, is not necessary to change the oscillators' synchrony profile. The identity of the neurons that constitute the synchronized population slowly drifts around the ring as a slowly moving traveling wave. As the drift's period is much larger than the oscillations' period, we can study the differences between the two domains, synchronized and unsynchronized (see ref. Abrams and Strogatz (2006) for details on the drift).

###  From a chimera state to hippocampal phase precession

**Figure 5:**
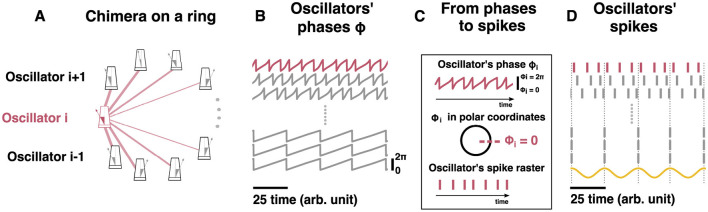
From a chimera state to hippocampal phase precession. **(A)** Schematic representation of the chimera state on a ring (see Equation 1 for details). For clarity, the coupling for oscillator *i* (pink or dark gray metronome for b/w printing) has been depicted (pink or dark gray edges for b/w printing). Edge thickness represents the weights of connections. **(B)** Time-series of different oscillators: some are asynchronous (top) and some are synchronous (bottom). **(C)** Cartoon explaining the transformation from the oscillators' phases to a putative “spike”: every time ϕ_*i*_ = 0, a spike occurs. **(D)** Oscillators' spike raster plot: panel **(B)** transformed into a raster plot. The sinusoidal curve (yellow) represents the macroscopic theta oscillation observed in an LFP. The theta oscillation is computed as the mean of cos(ϕ_*j*_), where ϕ_*j*_ corresponds to the phase from the synchronized oscillators. Dotted lines correspond to the peaks of the sinusoidal signal and to the spikes of the synchronized nodes. Model parameters: *A* = 0.95, β = 0.2 and *N* = 500, ρ = 1.

With the classical chimera dynamics reproduced, we investigated how to explicitly draw a mapping between the Kuramoto networks, specifically the ring network (Figure 5A), and hippocampal dynamics. Each neuron has more complex dynamics than those of a Kuramoto oscillator which is a simple oscillator where the frequency is integrated to arrive at the oscillator phase (Figure 5B). Specifically, neurons emit spikes when their inputs are sufficient to reach a threshold. Thus, each oscillator's continuous time-series was converted into spike trains via a Poincare Map. Each time any Kuramoto oscillator's phase reaches 2π, a “spike” is generated at the time that this occurred ($\theta_{j}\left(t^{*}\right)=2\pi$) as depicted in Figure 5C (see Methods for details). With a spike-generating Poincare map, the “spikes” generated by the chimera on a ring (Figure 5D) and for the two-population chimera (Figure 3) can be analyzed.

In order to measure phase-precession, an equivalent component to the hippocampal local field potential in the Kuramoto network is required. The hippocampal LFP is a macroscopic observable that is a complex synthesis of propagating action potentials, and synaptic activity. While there is some debate as to whether or not the LFP is reflective of underlying oscillations, or indeed organizes the timing of spikes, it is convenient to measure other oscillation frequencies (i.e., the oscillations of individual units) relative to the LFP (Buzsáki et al., 2012). During *in vivo* recordings, the hippocampal LFP is typically converted into a phase (for example with a Hilbert transform). Interneurons and sometimes pyramidal neurons lock to phases of the hippocampal LFP, while other pyramidal neurons fire at a slightly faster rate.

**Figure 6:**
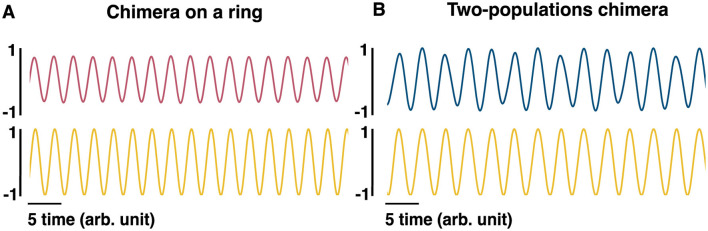
Phenomenological LFP models. **(A)** The pink (top) sinusoidal curve is computed as $\frac{1}{N}\overset{N}{\underset{i}{\sum }}\cos\phi_{i}$, where ϕ_*i*_ is the phase of oscillator *i* and can be regarded as an equivalent to the LFP for the chimera on a ring. The yellow (bottom) sinusoidal curve is computed as cosϕ_*s*_ where ϕ_*s*_ is the phase of one of the synchronized oscillators, and can also be regarded as an LFP. **(B)** The blue (top) sinusoidal curve is computed as $\frac{1}{n}\overset{n}{\underset{i}{\sum }}\cos\phi_{i}$+$\frac{1}{n}\overset{n}{\underset{j}{\sum }}\cos\gamma_{j}$, where ϕ_*i*_ and γ_*i*_ are the phases of oscillators *i* and *j*, respectively, and can be regarded as an equivalent LFP for the two-population chimera. The yellow (bottom) sinusoidal curve is computed as cosϕ_*i*_, given that ϕ_*i*_ belongs to the synchronized population.

Given the locking of synchronized sub-populations to the hippocampal LFP, a phenomenological LFP can be computed as follows: the cosine of the phase of each oscillator in the synchronized population is obtained (cosϕ_*j*_) and globally averaged over the synchronized population. The LFP can also be computed as the mean over all oscillators (both synchronized and unsynchronized): $\frac{1}{N}\overset{N}{\underset{i=0}{\sum }}\cos\phi_{i}$. Both methods of computing the LFP product qualitatively similar results (Figures 6A, B). We note that there are more direct, biophysically based models of LFPs considered in the literature (Mazzoni et al., 2015).

Interestingly, we observed phase advancement from the unsynchronized oscillators when compared to the synchronized ones (Figures 5D, C). While this is similar in principle to phase precession, where the unsynchronized pyramidal neurons fire slightly faster than the local-field-potential, the frequency ratio between the synchronized oscillators and the unsynchronized is different from those observed experimentally. For example, in Figure 5D, for every synchronized spike we get approximately three unsynchronized ones, which roughly gives us a ratio of ≈0.33. In the hippocampus, pyramidal neurons fire at approximately 9 Hz, while the theta oscillation observed in the LFP is approximately 8 Hz, which yields a a ratio of ≈ 0.88.

However, chimera states are solutions to coupled oscillator networks that are parameter dependent. Indeed, this is similar to limit cycles, chaotic solutions, or fixed points. The precise characteristics of all of these solutions depend on the chosen parameters for the underlying network. For example, in Panaggio et al. (2016), the chosen system parameters yield three unsynchronized spikes to one synchronized spike ratio as mentioned above. This ratio is also approximately the mean-phase velocity ratio between synchronized and unsynchronized populations. As another example, in Gu et al. (2013), it was found that the difference in mean-phase velocities can be very small, with only a 2% difference in the frequencies between the synchronized and unsynchronized populations. Finally, in Sawicki et al. (2017), the mean-phase velocity ratio is more intermediate in range, between 50%–100%. In some cases, the synchronized population can also oscillate faster than the unsynchronized population. All these differences in chimera dynamics arise from differences in the underlying models and model parameters. In the next section, we show that two well established chimera-capable models (Panaggio et al., 2016; Abrams and Strogatz, 2006) can yield phase-precession like spiking dynamics as in the hippocampus.

###  Changing the chimera state by changing the intrinsic frequency

Next, we investigated if the parameters in both models could be varied to both preserve the chimera state, and obtain a frequency ratio closer to that of hippocampal phase precession (≈0.88). Accomplishing this in both models would indicate that one can generically obtain hippocampal-like dynamics in chimera systems. To start, the intrinsic frequency parameter (ρ) was varied in Equations 1–3. This acts as the fundamental driving force for an oscillator and causes the oscillator to intrinsically oscillate when no coupling is present. Thus, it is directly comparable to the applied current *I* typically considered in neuron models as higher applied currents lead to faster neuronal oscillations.

As ρ was varied, the oscillating frequency for each oscillator was quantified as follows: the mean phase velocity Ω_*i*_ was computed for oscillator *i* to determine its frequency. As the driving frequency ρ interacts with the coupling in a non-trivial way, the frequencies must be computed numerically. For a given oscillator *i* and a given amount of time Δ*t*, the number of rotations around the origin (or equivalently, the number of spikes fired) was summed and multiplied by 2π (see methods and Equation 5 for details). This was then divided by Δ*t* to yield the rotations.

To see if the chimera dynamics could mimic hippocampal observations, we focused on the mean phase velocity ratio 〈Ω〉_*ratio*_. This ratio was computed as the average of the mean phase velocity ratio between the synchronized and unsynchronized populations as a function of ρ, as shown below (see Methods for details):


(18) $$\begin{array}{c} {\langle \Omega \rangle }_{ratio}=\frac{1}{m}\overset{j=m}{\underset{j=0}{\sum }}\frac{\Omega^{s}}{\Omega_{j}^{u}} \end{array}$$

**Figure 7:**
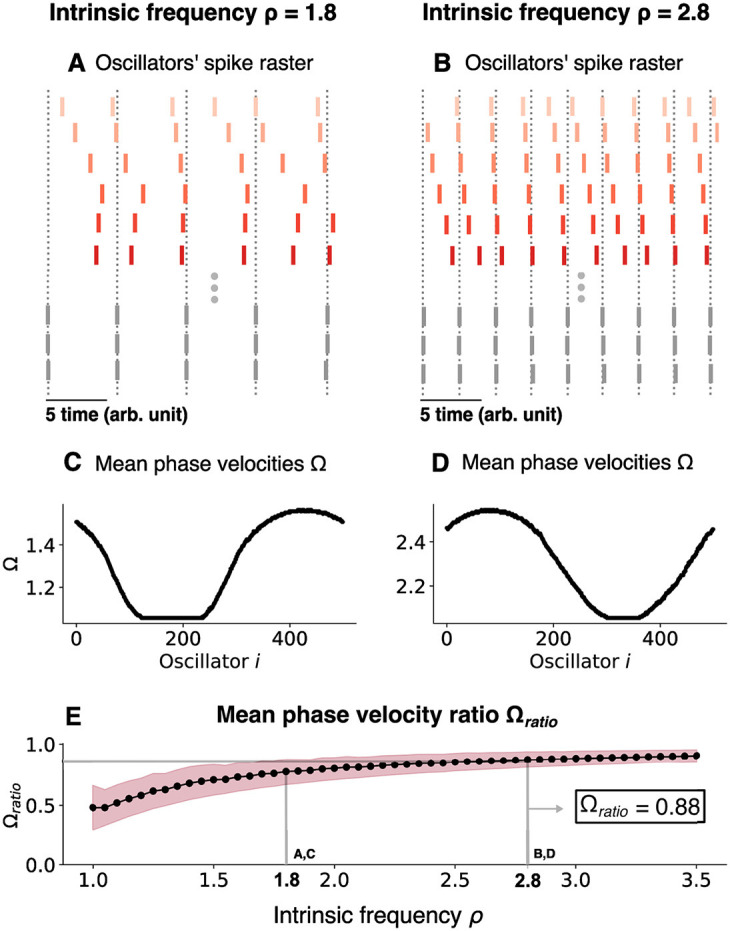
Changing the chimera state by changing the intrinsic frequency for the chimera on a ring. **(A)** Oscillators' spike raster plots for ρ = 1.8 and **(B)** ρ = 2.8, respectively. Note the theta sequences contained within a single oscillation cycle. Dotted lines correspond to the spikes of the synchronized nodes (gray ticks). **(C)** Mean phase velocity profile for ρ = 1.8 and **(D)** ρ = 2.8, respectively. **(E)** Mean phase velocity ratio 〈Ω〉_*ratio*_ as a function of the intrinsic frequency ρ. The pink region indicates the spread of the mean phase velocity ratio as computed by the standard deviation of 〈Ω〉_*ratio*_.

**Figure 8:**
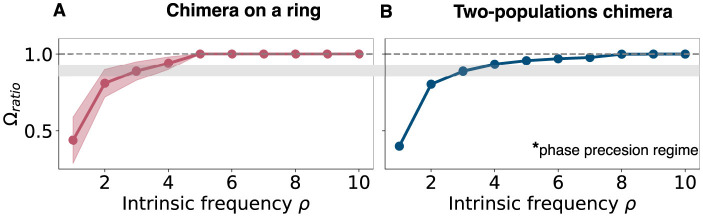
Mean phase velocity ratio for higher intrinsic frequencies. **(A)** Mean phase velocity ratio Ω_*ratio*_ for large values of the intrinsic frequency for the chimera on a ring. The pink region indicates the variation of the mean phase velocity ratio, since there isn't a unique value for the mean phase velocity for the unsynchronized group. It is computed as the standard deviation of $\frac{\Omega^{s}}{\Omega^{u}}$. **(B)** Mean phase velocity ratio Ω_*ratio*_ for large values of the intrinsic frequency for the two populations chimera. Gray region on both panels: it indicates where both systems have their phase precession regime, i.e., Ω_*ratio*_ = 0.88. Note that the two-population chimera has a well-defined synchronized and unsynchronized population, while the chimera on a ring system features oscillators that join and leave the synchronized population over long periods of time, leading to some variance in the estimate of the frequency-ratio that is not present for the two-population chimera.

As the intrinsic oscillation frequency (ρ) increases, the oscillation frequency of both the synchronized and unsynchronized oscillators in the coupled network increases, but the frequency difference between the synchronized and unsynchronized domains decreases. This was quantified for ρ = 1.8 (Figures 7A, C) and ρ = 2.8 (Figures 7B, D) and, more generally, for the mean phase velocity ratio as a function of ρ (Figure 7E). As ρ was varied, Ω^*u*^ varied over a range which was bounded by a minimum Ω_*min*_ and a maximum value Ω_*max*_. For ρ = 1.8, (Ω_*min*_, Ω_*max*_) = (1.056, 1.565) while for ρ = 2.8, they increase to (Ω_*min*_, Ω_*max*_) = (2.055, 2.545) and we achieve 〈Ω〉_*ratio*_≈0.88 for that value (Figure 7E). As ρ is increased further past this value, the ratio slowly increases until the chimera state collapses and all oscillators synchronize (Figure 8).

**Figure 9:**
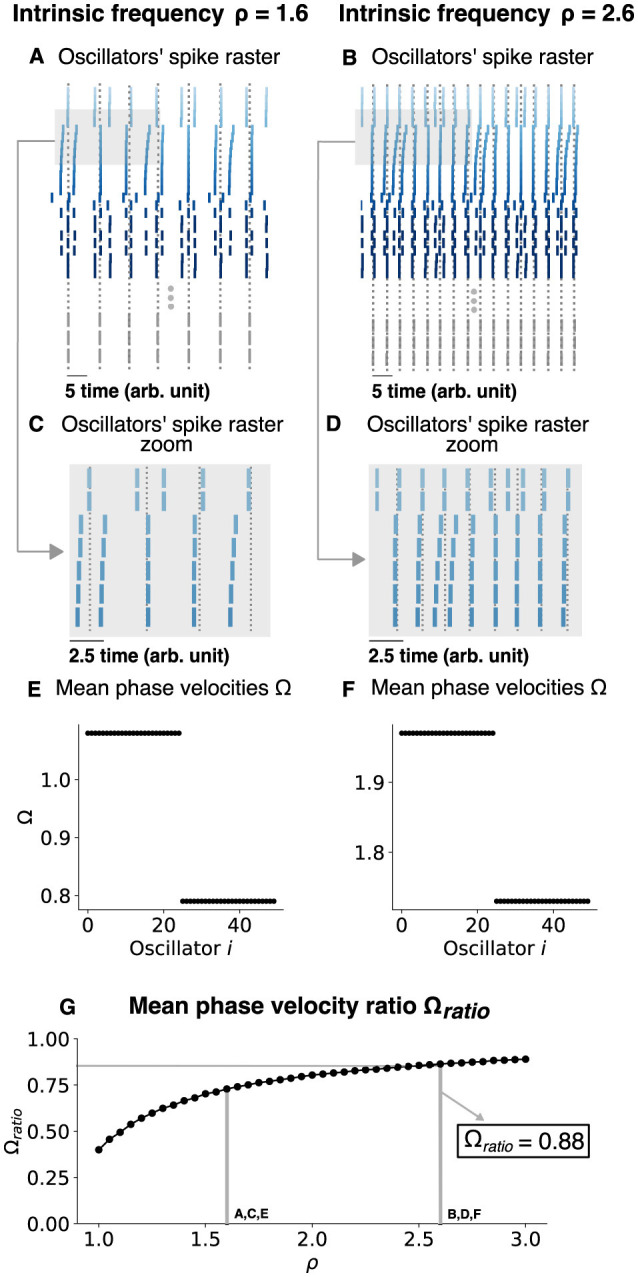
Changing the chimera state by changing the intrinsic frequency for a two-population chimera. **(A, B)** Oscillators' spike raster plots for ρ = 1.8 and ρ = 2.8, respectively. Dotted lines correspond to the spikes of the synchronized nodes (gray ticks). **(C, D)** Respectively, zoom in on panels **(A, B)** (light gray box). **(E, F)** Mean phase velocity profile for ρ = 1.8 and ρ = 2.8, respectively. **(G)** Mean phase velocity ratio Ω_*ratio*_ in function of the intrinsic frequency ρ. Here, *n* = 25 oscillators were used for each population.

Next, we tested if this was a generic response by considering the two-population chimera model (Figure 9). Once again, we found that the 〈Ω〉_*ratio*_≈0.88 can occur for a specific ρ due to the slow gradual increase in 〈Ω〉_*ratio*_ as a function of ρ. Thus, the phase precession regime of classical chimera models is seemingly robust and generic.

Finally, we investigated what the net impact of the coupling was. That is, we considered how the mean phase velocity for both domains (synchronized and unsynchronized) and for both network topologies compares to the mean phase velocity of an uncoupled oscillator. In the latter case, the mean-phase velocity is given by


$$\begin{array}{c} \Omega_{uc}=2\pi \rho \end{array}$$

**Figure 10:**
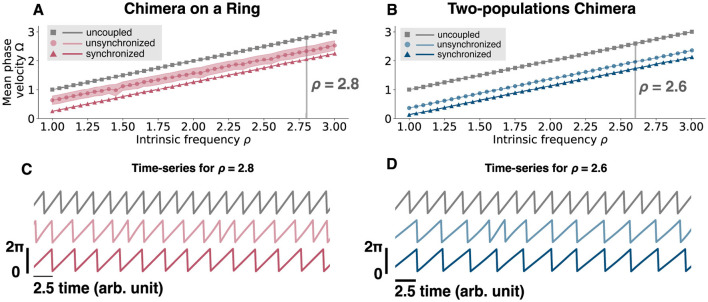
Mean phase velocity for an uncoupled oscillator and for different intrinsic frequencies. **(A)** Mean phase velocity in function of the intrinsic frequency ρ for an uncoupled oscillator, i.e., $\frac{d\phi_{i}}{dt}=\rho$ (gray squares), for the unsynchronized oscillators (light pink circles), and for the synchronized oscillators (pink triangles) of the chimera on a ring. For the unsynchronized oscillators the mean phase velocity is computed as the mean of Ω^*u*^, since we get a different $\Omega_{i}^{u}$ for each oscillator *i*. The light pink region is computed as the standard deviation of Ω^*u*^. **(B)** Mean phase velocity in function of the intrinsic frequency ρ for an uncoupled oscillator, i.e. $\frac{d\phi_{i}}{dt}=\rho$ (gray squares), for the unsynchronized population (light blue circles), and for the synchronized population (blue triangles) of the two-populations chimera. **(C)** Time-series for ρ = 2.8 for the three different cases, uncoupled (gray, top), unsynchronized (light pink, middle) and synchronized (pink, bottom) for the chimera on a ring. **(D)** Time-series for ρ = 2.8 for the three different cases, uncoupled (gray, top), unsynchronized (light blue, middle), and synchronized (blue, bottom) for the two-populations chimera.

Interestingly, regardless of the network topology, the net effect of the coupling was always inhibitory: The oscillators fire at a faster frequency when uncoupled, rather than when coupled into a chimera state in both network topologies (Figure 10).

**Figure 11:**
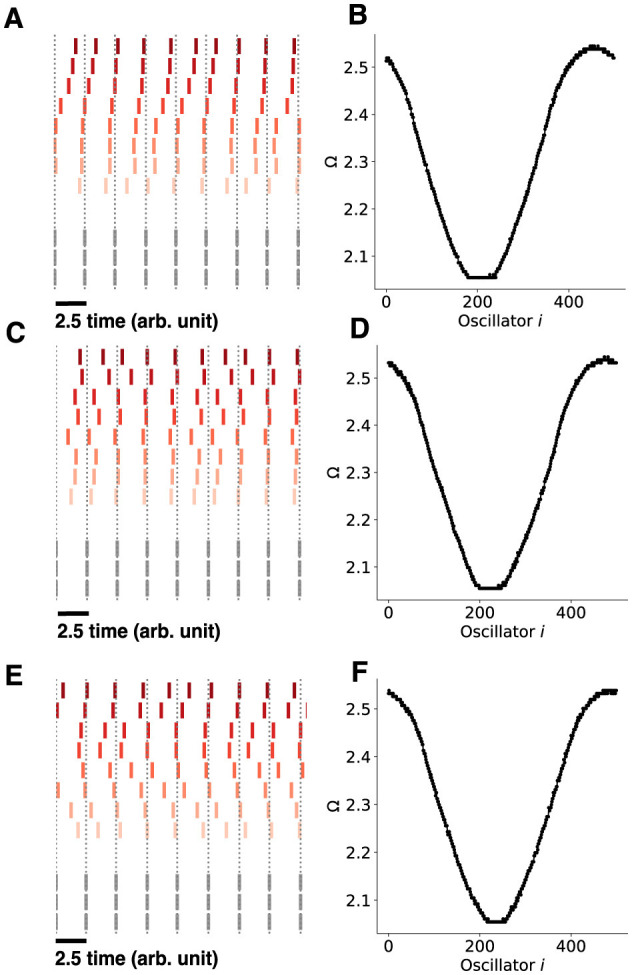
Chimera states with noise for the chimera on a ring. A white noise process is injected into each oscillator with different noise standard deviations. **(A)** The chimera on a ring consists of 500 oscillators, each receiving a white noise process with mean 0 and standard deviation σ = 0.0071, *D* = 2.5 × 10^−5^. **(B)** The mean phase velocities of the Kuramoto oscillators. **(C, D)** identical as in (A)–(B), only with σ = 0.001, *D* = 5 × 10^−5^. **(E, F)**, identical as in **(A, B)**, only with σ = 0.0014, *D* = 0.0001. Note that the sequential content in all cases was preserved in the non-synchronized population, only with larger amounts of jitter for larger values of σ.

Finally, we investigated the impacts of noise on the Kuramoto system (Figure 11) in the phase precessing regime. We found that injecting white noise into each oscillator for the chimera on a ring, with mean 0 and standard deviation σ did not substantially impact the results, with similar phase precession dynamics and mean-phase velocities when σ was small.

###  Phase precession in a chimera-trained spiking neural network

Chimera dynamics in networks of Kuramoto oscillators with different network topologies can be altered by changing one parameter, the intrinsic driving frequency, to mimic a hippocampal-like phase precession regime. Despite the general nature of these results, the Kuramoto-oscillator network is phenomenologically different from the neurons and synaptic connections in the hippocampus in addition to having the property that all of the oscillators are homogeneous. Thus, we sought to determine if embedding a chimera state into a spiking-neural-network would still yield hippocampal phase precession, and a global theta-oscillation.

**Figure 12:**
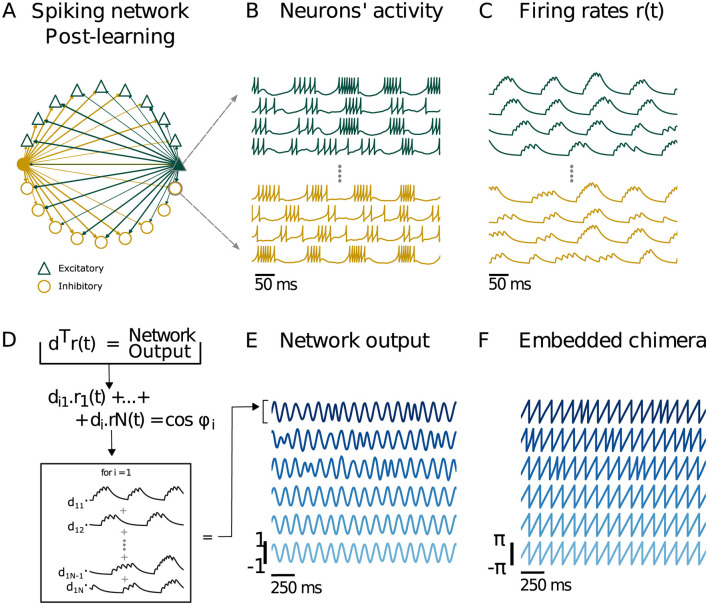
Training a spiking neural network to output a chimera state from the two-population chimera. **(A)** Spiking neural network. Each node represents either an excitatory (green or dark gray triangle for b/w printing) or inhibitory (yellow or light gray circle for b/w printing) neuron. For clarity, only the connections for two neurons (filled triangle and circle) are depicted. The network respects Dale's law: an excitatory (inhibitory) neuron will only excite (inhibit) its connections, regardless of the neuron target type. As a result, excitatory (inhibitory) neurons just have green or dark gray for b/w printing (yellow or light gray for b/w printing) outgoing connections. **(B)** Voltage traces for excitatory (green or dark gray triangle for b/w printing) and inhibitory (yellow or light gray round for b/w printing) neurons. **(C)** Firing rates ***r***(***t***) obtained from filtering the spikes with a two double exponential filter, see equations for details. **(D)** The network output is given by ***d***^⊤^*r*(*t*), which is a *s* x *n*_*t*_ matrix (*s* is the total number of supervisors). Each network output column *i* is *n*_*t*_ time units long and is a linear combination of the firing rates *r*_1_(*t*), ⋯ , *r*_*N*_(*t*) with *d*_*iN*_ as coefficients. **(E)** Network output $\cos\hat{\phi }\left(t\right)$ and $\cos\hat{\rho }\left(t\right)$. **(F)** Embedded Chimera from network output: $\hat{\phi }\left(t\right)=\frac{\sin\hat{\phi }\left(t\right)}{\cos\hat{\phi }\left(t\right)}$ and $\hat{\rho }(t)=\frac{\sin\hat{\rho }(t)}{\cos\hat{\rho }(t)}$. We recover the two-populations chimera (where we had *n* = 3 oscillators per population).

**Figure 13:**
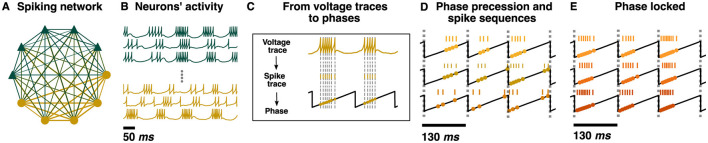
Phase precession in a chimera-trained spiking neural network with the two population chimera. **(A)** A cartoon schematic of the spiking neural network with Dale's law. Each node represents either an excitatory (green or dark gray triangle for b/w printing) or inhibitory (yellow or light gray circle for b/w printing) neuron. The network respects Dale's law: an excitatory (inhibitory) neuron will only excite (inhibit) its connections, regardless of the neuron target type. As a result, excitatory (inhibitory) neurons only have green or dark gray for b/w printing (yellow or light gray for b/w printing) outgoing connections. Edge thickness represents the weight of each connection. **(B)** Voltage traces for excitatory (green or dark gray triangle for b/w printing) and inhibitory (yellow or light gray round for b/w printing) neurons. **(C)** From top to bottom: the voltage trace of an inhibitory neuron from the spiking neural network, its correspondent spike sequence and its projection to the phase $\hat{\phi }$ of the synchronized population (black trace), which represents the theta oscillation. Gray dotted lines mark every time the voltage reaches its peak *v* = 30 mV and a spike is generated. **(D)** Example of phase precession and spike sequences from three inhibitory neurons. For each neuron, the spike sequence and its projection into the phase $\hat{\phi }$ is plotted. Gray dotted lines mark every time $\hat{\phi }=0$. **(E)** Example of three phase locked inhibitory neurons. Gray dotted lines mark every time $\hat{\phi }=0$.

A chimera state can be “embedded” in a recurrent neural network by training the network to output a chimera, as seen in (Masoliver et al. 2022). To test if such an embedding was applicable in a spiking network, we trained a spiking neural network using the FORCE method (Sussillo and Abbott, 2009; Nicola and Clopath, 2017) to output the two-population chimera, described by Equations 2, 3. This network was constrained with Dale's law, with a proportion of the neurons being excitatory, and the rest inhibitory. Initially, the individual neurons (modeled using the Izhikevich model, see Methods for details) are sparsely connected [to support the learning process (Sussillo and Abbott, 2009)] with a set of static weights *Gω*_0_ which initiate the neurons' rate *r*(*t*) into a high-dimensional chaotic regime. During the training period a second set of weights *Qη**d***^*T*^ is added to *Gω*_0_ and changes the connections between neurons such that the network's output (defined as ***d***^*T*^*r*) equals the desired dynamics. The desired dynamics or supervisor are cosines of the phases of a two-population Kuramoto oscillator network in the chimera regime. At each time step, ***d*** is updated using the Recursive Least Squares (RLS), which minimizes the sum-squared difference between the network output and the two-population chimera. The network has learned when for a fixed value of ***d*** it is able to mimic the desired chimera dynamics (Figure 12). A specific example is shown in Figure 13. We remark that while the chimera state supervisors have homogeneous oscillators, the trained neurons whether in a rate or spiking network are heterogeneous, as they receive a combination of randomly generated, and trained weights which alters their activity levels and how they encode the chimera dynamics.

In order to assess if the individual neurons of the spiking network show phase precession, the voltage traces were transformed into phases (Figure 13C). The spike times were transformed into phases by using a linear interpolation to approximate the phase at each spike time with the phase of one of the synchronized components of the network output ${\hat{\phi }}_{syn}\left(t\right)$. We found that phase precession occurred generically for many of the neurons sampled, where neurons displayed decreasing burst phases based on subsequent cycles (Figure 13D). However, some of the neurons were primarily phase locked (Figure 13E).

## Discussion and conclusions

Since their discovery, chimeras have been extensively modeled, applied, and recently experimentally realized in the study of complex oscillatory systems (Gu et al., 2013; Panaggio and Abrams, 2015; Sawicki et al., 2017; Davidsen, 2018; Parastesh et al., 2020; Lau et al., 2023). More recently, attempts have been made to link them directly to brain dynamics, using largely modeling studies and different coupling topologies (Majhi et al., 2019). This includes chimeras in oscillating brain networks (Chouzouris et al., 2018; Bansal et al., 2019), three-dimensional chimeras in spiking neuronal networks (Kasimatis et al., 2018), chimeras in heterogeneous networks (Laing, 2009, 2017) and the robust emergence of chimeras in recurrent neural networks (Masoliver et al., 2022) as well as limited experimental studies (Lainscsek et al., 2019). Yet, their potential functional role in brain dynamics has remained largely elusive. Chimeras have been hypothesized to be the dynamical state dolphins, birds, and other animals that need to navigate over large ranges in 3-dimensions utilize to sleep, where half the brain is in a synchronized sleep state while the other half is in an asynchronous awake state (Parastesh et al., 2020). Similarly, chimeras might potentially play a role in memory consolidation related to REM and non-REM sleep (Curic et al., 2023).

Here, we utilized computational modeling to test the hypothesis that the hippocampal phase precession regime that occurs during the hippocampal theta oscillation may be a chimera state. By modifying the intrinsic frequency parameter in the Kuramoto oscillators exhibiting a classical chimera, and using a spike-generating Poincare map, we found that chimera dynamics readily produced theta-phase precession-like observations over a range of values. The oscillators in the asynchronous group fired slightly faster (~ 1 Hz) than those in the synchronous group, resulting in theta phase precession. The spikes generated by these oscillators also displayed theta-sequence-like activity. We found that the net coupling in both the chimera-on-a-ring and two-population chimera was inhibitory, as deactivating the coupling resulted in a higher mean-phase-velocity than with the coupling in place. Finally, we embedded a chimera state into a spiking neural network of Izhikevich neurons with Dale's Law constraining the connection weights through FORCE training. Despite the embedded nature of the chimera, at the micro-scale, the spiking neurons still displayed phase-precession (asynchrony) and phase locking (synchrony), the observable features of the chimera. This is despite the heterogeneity in the coupling the neuron's display. We note that while FORCE training is not a biologically plausible learning algorithm, it can find biologically plausible solutions to the connection weights that can lead to specific network behaviors (Sussillo and Abbott, 2009). One limitation of this current work is the use of even ratios of 50/50 excitatory/inhibitory neurons, which is common in spiking neural network implementations of reservoir computing (Nicola and Clopath, 2019, 2017). This is not a realistic assumption of the current work, as there is an 80/20 split of excitatory to inhibitory neurons in the hippocampus (Freund and Antal, 1988). To the best of our knowledge, this study is the first to postulate and test the hypothesis that the hippocampal phase precession is a chimera state.

Interestingly we found that the synchronized and unsynchronized population(s) can drift, and thus the designation as being part of the synchronized and unsynchronized population is non-static while the global chimera state persists. This feature is generic to many chimera models, especially in chimera models involving 3-dimensional structures (Panaggio and Abrams, 2013, 2015; Maistrenko et al., 2015; Lau and Davidsen, 2016). Most importantly, this is consistent with the fact that phase precessing pyramidal neurons are not fixed and change their dynamics over time (O'keefe and Burgess, 2005). This distinguishes the chimera hypothesis fundamentally from other hypotheses for the generation of hippocampal phase precession, where the phase-precession effect can be fixed by either the local or global connectivity (e.g., Nicola and Clopath, 2019; Chadwick et al., 2016; Bose et al., 2000). Indeed, this is the intrinsic difference between chimera dynamics, and other models of phase precession: chimeras allow considerable flexibility in which neurons are phase precessing dependent on changing the initial conditions or external inputs or perturbations. However, we do not discount the possibility that prior models of phase precession may exhibit latent chimera dynamics.

Further, we remark that some chimera states may be “super-transients” (Wolfrum and Omel'chenko, 2011), which are not asymptotically stable states but reflect a long, but ultimately unstable state on the route to a stable one (either asynchrony or synchrony). Super-transient dynamics can occur in non-linear systems for a very long time before convergence to the eventual stable state. We note that the work considered here is compatible with super-transients, as the hippocampal theta oscillation is not an indefinite state, but is stopped under a variety of conditions like slow locomotion or entering into slow-wave sleep states (Buzsáki, 2015).

**Figure 14:**
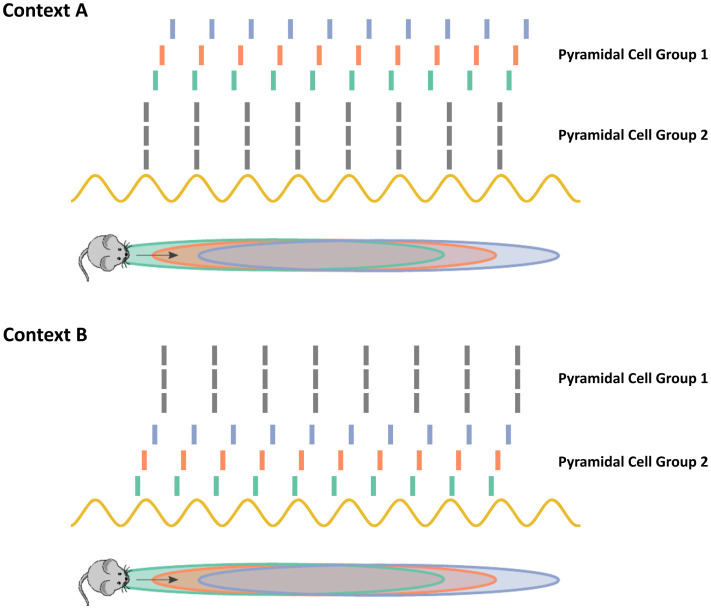
Chimera dynamics allow the hippocampus to select subsets of neurons for phase-precession based coding. When a mouse enters into a novel environment or context, as in contexts A and B in the cartoon above, subsets of phase precessing neurons encode the animals navigational trajectory. How the hippocampus can flexibly select different subsets of neurons remains an open challenging. In the chimera hypothesis, the synchronous and asynchronous states are both stable attractors. The context cues act to initialize the system within the basin of attraction for the different subsets of neurons. Depending on the specifics of the chimera coupling, the distribution and long-term stability of the asynchronous/synchronous populations may differ. For example, in the two population chimera model, the asynchronous/synchronous populations are equal in size with stable dynamics, while in the chimera on a ring a subset of the neurons synchronize and the synchronous subset slowly drifts along the ring as neurons join and leave the synchronous group.

It remains an open question how the hippocampus can utilize chimera dynamics to encode memories. One intriguing possibility is that chimera dynamics produce local stability or local transient stability of many possible subsets of pyramidal neurons in the asynchronous, phase precessing state as shown in Figure 14. The context that the animal is in provides a series of cues that ultimately become translated into neuronal firing states in the hippocampal circuit. This initial neuronal firing can be thought of as the initial state of an oscillator network. Depending on which initial state the hippocampal system is in, it will fall into the basin of attraction for a specific configuration of synchronous and asynchronous subpopulations (Figure 14). This allows for the flexible selection of different populations of neurons to encode potentially many different contexts, depending on the specifics of the chimera in question. For example, the cues in context A may map to an initial state where pyramidal cell group 2 is nearly synchronized. Then, as the chimera solution is locally stable, pyramidal cell group 1 begins precessing in phase. Context B however may produce cues that map to an initial state where pyramidal cell group 1 is more synchronized, thereby leading to an alternate synchronous/asynchronous division of cell groups. It is also possible that the relationship between cues and initial states of the chimera system is learned. The presynaptic inputs into the hippocampal circuit, possibly from the entorhinal cortex learn to initialize the system into different chimera configurations.

Chimera states have proven to be ubiquitous and robust in nature, whether implemented as collections of simple pendulums or metronomes, or in the underlying dynamics behind chemical reaction equations. However, the heterogeneity and noise present in biological systems may destabilize these dynamical states. Here, we show that Chimera states may contribute to hippocampal phase-precession, and possibly present the first biological chimera state observable at a cellular level.

## Data Availability

Publicly available datasets were analyzed in this study. This data can be found here: https://github.com/mariamasoliver/link-phase-precession-and-chimeras.

## Supplementary material

Supplementary Video S1 A simulation of the two population chimera state as in Equation 3.

Supplementary Video S2 A simulation of the chimera state on a network of Kuramoto oscillators coupled on a ring as in Equation 1.
